# Graphene oxide suppresses the growth and malignancy of glioblastoma stem cell-like spheroids via epigenetic mechanisms

**DOI:** 10.1186/s12967-020-02359-z

**Published:** 2020-05-14

**Authors:** Xu Wang, Wenjuan Zhou, Xian Li, Jun Ren, Guangyu Ji, Jingyi Du, Wenyu Tian, Qian Liu, Aijun Hao

**Affiliations:** 1 Key Laboratory for Experimental Teratology of Ministry of Education, Shandong Key Laboratory of Mental Disorders, Department of Anatomy and Histoembryology, School of Basic Medical Sciences, Cheeloo College of Medicine, Shandong University, 44#, Wenhua Xi Road, Jinan, 250012 Shandong China; 2 Department of Foot and Ankle Surgery, The Second Hospital, Cheeloo College of Medicine, Shandong University, Jinan, 250012 Shandong China

**Keywords:** Graphene oxide, Glioblastoma stem-like cells, Epigenetics, Cell differentiation

## Abstract

**Background:**

Glioblastoma stem-like cells (GSCs) are hypothesized to contribute to self-renewal and therapeutic resistance in glioblastoma multiforme (GBM) tumors. Constituting only a small percentage of cancer cells, GSCs possess “stem-like”, tumor-initiating properties and display resistance to irradiation and chemotherapy. Thus, novel approaches that can be used to suppress GSCs are urgently needed. A new carbon material—graphene oxide (GO), has been reported to show potential for use in tumor therapy. However, the exact effect of GO on GSCs and the inherent mechanism underlying its action are not clear. In this study, we aimed to investigate the usefulness of GO to inhibit the growth and promote the differentiation of GSCs, so as to suppress the malignancy of GBM.

**Methods:**

In vitro effects of GO on sphere-forming ability, cell proliferation and differentiation were evaluated in U87, U251 GSCs and primary GSCs. The changes in cell cycle and the level of epigenetic modification H3K27me3 were examined. GO was also tested in vivo against U87 GSCs in mouse subcutaneous xenograft models by evaluating tumor growth and histological features.

**Results:**

We cultured GSCs to explore the effect of GO and the underlying mechanism of its action. We found, for the first time, that GO triggers the inhibition of cell proliferation and induces apoptotic cell death in GSCs. Moreover, GO could promote the differentiation of GSCs by decreasing the expression of stem cell markers (SOX2 and CD133) and increasing the expression of differentiation-related markers (GFAP and β-III tubulin). Mechanistically, we found that GO had a striking effect on GSCs by inducing cell cycle arrest and epigenetic regulation. GO decreased H3K27me3 levels, which are regulated by EZH2 and associated with transcriptional silencing, in the promoters of the differentiation-related genes GFAP and β-III tubulin, thereby enhancing GSC differentiation. In addition, compared with untreated GSCs, GO-treated GSCs that were injected into nude mice exhibited decreased tumor growth in vivo.

**Conclusion:**

These results suggested that GO could promote differentiation and reduce malignancy in GSCs via an unanticipated epigenetic mechanism, which further demonstrated that GO is a potent anti-GBM agent that could be useful for future clinical applications.

## Background

GBM is the most common and aggressive primary brain cancer [1]. Previous studies have demonstrated that GBMs harbor subpopulations that are in different genetic and epigenetic states, including GSCs [2]. GSCs, which form spheres with self-renewing and tumor-propagating capacity, are capable of efficiently growing in xenograft models [3]. It has been suggested that GSCs contribute to the rapid growth, and self-renewal of glioblastoma, and likely drive the onset of tumor growth, therapeutic resistance, and tumor recurrence [4–6]. The existing literature is still insufficient and new research and treatment strategies focusing on GSCs are urgently needed.

Graphene is a two-dimensional carbon allotrope with many unique properties, such as excellent electroconductivity, high specific surface area and good biocompatibility [7]. Graphene and its oxidized forms, including graphene oxide (GO), have shown great potential for biological and medical applications [8, 9]. Previous studies have shown that GO could promote the adhesion and differentiation of mesenchymal stem cells cultured without special differentiation media and facilitate the differentiation of neural stem cells into neural-like cells [10, 11]. In glioblastoma, it was revealed that graphene nanoparticles induced cell death mostly via the apoptosis pathway, and both GO and reduced graphene oxide decreased the volume and weight of tumors [12, 13]. However, the exact effects of GO on GSCs and the underlying mechanism are not clear.

Cancer malignancy is dynamically affected by epigenetic mechanisms. Polycomb repressive complex 2 (PRC2) proteins play essential regulatory roles in cell-fate decisions via the trimethylation of lysine 27 on histone H3 (H3K27me3). It is clear that the enhancer of zeste 2 (EZH2) is the key methyl transferase that directly regulates the H3K27me3. In a wide variety of cancers, including GBM, EZH2 is highly expressed, and its expression is positively correlated with tumor malignancy and invasiveness [14]. It was demonstrated that the overexpression of EZH2 could lead to the inhibition of several tumor suppressors, including RKIP, p19 and p57 [15, 16]. However, this previous study ignored the potential influence of GO on the epigenetic system, and the underlying molecular mechanism of the anti-GSC effect of GO is still unclear. Therefore, our research aims to explore this phenomenon and attempt to elucidate the mechanism by which GO regulates GSCs.

In this study, we attempted to confirm the effect of GO on GSCs, and determine whether the mechanism underlying the effects of GO involved histone modification. We examined the effect of GO on GSCs by measuring the expression levels of various genes related to proliferation, differentiation and the cell cycle. Finally, we attempted to determine the mechanism underlying the effect of GO on GSCs. This is the first time that the biological effects and the epigenetic mechanisms utilized by GO in GSCs has been explored.

## Methods

### Cell culture and isolation of GSCs

The human glioblastoma cell lines U87 and U251 were used for study. They were purchased from the American Type Culture Collection (ATCC) and cultured in DMEM high-glucose medium (Thermo Fisher Scientific, USA), supplemented with 10% fetal bovine serum (Gibco, USA), 100 U/ml penicillin and 100 μg/ml streptomycin (Millipore, USA) at 37 °C in 100 mm dishes in a humidified incubator with 5% CO_2_. When the cells reached 90% confluence, the cells were digested with 0.25% trypsin (Gibco, USA) for 1 min and resuspended in serum-free medium composed of DMEM/F‑12 (Gibco, USA), 2% B27 (Gibco, USA), 20 ng/ml EGF (Invitrogen, USA), and 20 ng/ml bFGF (R&D, USA). The cells were cultured at 37 °C in 5% CO_2_ for 7 days to enrich for GSCs. Subsequently, the GSCs were treated with different concentrations of GO.

Patient-derived GSCs, GBM#BG5 was established from GBM surgical specimens at the K. G. Jebsen Brain Tumour Research Centre, Department of Biomedicine, University of Bergen and was kindly gifted by Prof. Rolf Bjerkvig (University of Bergen, Bergen, Norway). The cells were cultured in Neurobasal™ Medium (Gibco/Thermo Fisher Scientific; Waltham, MA), supplemented with B27 supplement, 20 ng/mL bFGF, 20 ng/mL EGF, and 1% GlutaMAX™ (Gibco/Thermo Fisher Scientific; Waltham, MA).

### Characterization of GO

GO was purchased from Sigma-Aldrich (Saint Lou, USA). GO was produced using the modified Hummers method and dispersed in water at a concentration of 2 mg/ml. The GO flakes contained carboxyl, epoxide, hydroxide and ketone groups, and they were maintained in a chloride-free sate using dialysis purification. The mean diameter of the GO monolayer sheet was 22 μm, and most of the sheets were smaller than 50 μm in diameter. GO flakes with diameters between 0.2 μm and 20 μm were able to target cells [17]. Prior to the treatment of the cells, GO were sonicated for 30 min and diluted to an appropriate concentration in culture medium.

### Cell viability assay

An MTT assay (Solarbio) was used to measure the cell proliferation of GSCs. The 96-well plates were pretreated with poly-l-Lysine (Sigma-Aldrich). Approximately 8 × 10^3^ GSCs were seeded in each well of a 96-well plate, to which was added an appropriate concentration of GO. After treatment for 2, 4, and 6 days, 10 μM MTT was added to each well and incubated at 37 °C for 2 h. Then, the culture medium in each well was replaced with 150 μL of DMSO solution. The absorbance at a wavelength of 490 nm was measured by a microplate autoreader.

### Western blot analysis

The GSCs were lysed with RIPA buffer (Beyotime Institute of Biotechnology, Shanghai, China) containing protease and phosphatase inhibitors for 30 min at 4 °C. Then the supernatants were collected after centrifugation at 12,000 rpm at 4 °C for 15 min. The supernatants were mixed with loading buffer (Boster, Wuhan, China), and equal amounts of protein were separated by sodium dodecyl sulfate–polyacrylamide gel electrophoresis, and then transferred to PVDF membranes. The PVDF membranes were blocked and incubated with the primary antibodies at 4 °C overnight. The primary antibodies were used at the following dilutions: rabbit anti-β-actin (1:2000; #4970, CST), mouse anti-β-III tubulin (1:1000; #4466, CST), rabbit anti-GFAP (1:1000; BA0056, Boster), mouse anti-sox2 (1:1000; ab171380, Abcam), rabbit anti-CD133 (1:1000; ab19898, Abcam), rabbit anti-H3 (1:1000; #4499, CST), rabbit anti-OCT4 (1:1000; ab18976, Abcam), rabbit anti-acetyl-H3 (1:1000; #06-599, Millipore), rabbit anti-H3K27me3 (1:1000; #9733, CST), mouse anti-H3K9me3 (1:1000; #5327, CST), rabbit anti-EZH2 (1:1000; #5246, CST). After washing with TBST, the membranes were incubated with HRP-conjugated goat anti-mouse or goat anti-rabbit secondary antibodies at room temperature. The antibody labeling was detected using an enhanced chemiluminescence reagent (Merck Millipore). The protein bands were analyzed using ImageJ.

### RNA isolation and real-time quantitative PCR

The GSCs were harvested after treatment with GO, and TRIZOL reagent (Invitrogen, Carlsbad, CA, USA) was used to extract the total RNA from the GSCs. Then, cDNA was synthesized using a RevertAid™ First Strand cDNA Synthesis Kit (Thermo Fisher Scientific). Quantitative PCR was performed in triplicate using a real-time PCR system with Realtime PCR Master Mix (TOYOBO CO., Ltd., Japan). The expression level was normalized to the expression of GAPDH and calculated using the comparative 2^−ΔΔ^Ct method. The primer sequences are listed in Table 1.

**Table 1:** The primer sequence used in RNA based PCR

GENE	Forward primer	Reverse primer
SOX2	ATGGGTTCGGTGGTCAAGTC	CGCTCTGGTAGTGCTGGGACA
CD133	AGTCGGAAACTGGCAGATAGC	GGTAGTGTTGTACTGGGCCAAT
GAPDH	CTGGGCTACACTGAGCACC	AAGTGGTCGTTGAGGGCAATG
CDK4	ATGGCTACCTCTCGATATGAGC	CATTGGGGACTCTCACACTCT
CDK6	TCTTCATTCACACCGAGTAGTGC	TGAGGTTAGAGCCATCTGGAAA
CYCLIND1	CAATGACCCCGCACGATTTC	CATGGAGGGCGGATTGGAA
EZH2	AATCAGAGTACATGCGACTGAGA	GCTGTATCCTTCGCTGTTTCC
KDM6A	TTCCTCGGAAGGTGCTATTCA	GAGGCTGGTTGCAGGATTCA
KDM6B	CGCTGCCTCACCCATATCC	ATCCGCGACCTCTGAACTCT

### Flow cytometry (FCM) analysis

The GSCs were collected and washed with cold PBS three times. Then, the cells were fixed and permeabilized with 75% alcohol for 24 h. The GSCs were stained with propidium iodide (PI) (Beyotime Institute of Biotechnology, Shanghai, China). After incubation in the dark for 15 min, the stained cells were analyzed using a flow cytometer and counted using ModFit software. The cell cycle results indicated the exact distribution of the cells in the G0–G1, S, and G2-M phases.

### Immunofluorescence staining

GSCs were incubated on polylysine-coated coverslips. After 48 h of treatment with GO, the cells on the coverslips were treated with 4% paraformaldehyde for 30 min. The coverslips were washed with PBS, followed by permeabilization with 0.3% Triton X-100 for 30 min at room temperature. The coverslips were blocked with 10% goat serum. We performed incubation with anti-CD133 (1:200; PB0168, Boster) and anti-GFAP (1:200; BA0056, Boster) antibodies overnight, which was followed by incubation with secondary antibody for 1 h. The nuclear DNA was labeled with DAPI. Representative images were obtained with an IX71 Olympus fluorescence microscope.

### TUNEL staining and EdU labeling analyses

Apoptotic cell death was detected according to the protocol that was included with the TUNEL kit (Fluorescein In Situ Apoptosis Detection Kit, KeyGEN BioTECH, China). The GSCs grown on the polylysine-coated coverslips were treated with GO, and then the coverslips were fixed with 4% paraformaldehyde for 30 min. The coverslips were used for TUNEL staining. After labeling, images were acquired by fluorescence microscopy.

The EdU labeling assays were performed using the Cell-Light EdU Apollo 488 In Vitro kit (RiboBio, Guangzhou, China). GSCs were treated with 50 μM EdU for 2 h at 37 °C according to the manufacturer’s protocol. Then cells were fixed with 4% paraformaldehyde, permeabilized with 0.4% Triton X-100, and incubated with Apollo^®^ reagent. The nuclear DNA was labeled with DAPI and representative images were obtained. The number of EdU- or DAPI-positive cells was counted with ImageJ.

### Chromatin immunoprecipitation

Chromatin immunoprecipitation (ChIP) was conducted using an EZ-ChIP kit (Merch Millipore). GSCs were treated with 1% formaldehyde, which served as a cross-linking agent, and were then incubated with glycine at room temperature for 10 min to terminate the cross-linking reaction. The GSCs were sonicated to shear the DNA into chromatin fragments of ~ 200–500 bp. The supernatants were incubated overnight with H3K27me3 antibody or control antibody (anti-IgG). Then, the supernatants were subject to washing, elution and cross-link reversal processes that were performed according to the manufacturer’s instructions. The purified DNA fragments were subjected to real-time PCR. The primer sequences are listed in Table 2.

**Table 2:** The primer sequence used in ChIP-qPCR

GENE	Forward primer	Reverse primer
GFAP#1(ChIP)	TTGGAAAGCAGGTCAGAGG	GGTGGCTCATGCTTGTAATC
GFAP#2(ChIP)	GACTCACCTTGGCACAGAC	ATAGAGCCTTGTTCTCCACC
TUJ1#1(ChIP)	CCTGGGAAATGCTTGATGT	CAGAGGAAATGGAGGTGGTC
TUJ1#2(ChIP)	GGGGAACGGAGGAGGACAT	CATTGGAGCAGACGGAGTG

### In vivo tumor formation assay

Four-week-old BALB/c-nude mice were purchased from Vital River Laboratories (Beijing, China). All mice were assigned randomly to one of two groups (n = 5 each group). GSCs were collected; concentration of the resuspended cells was 1 × 10^7^ cells/mL. Then, the 1 × 10^6^ untreated GSCs or GO-treated GSCs were injected subcutaneously into the right side of the posterior flank of each mouse. Tumor growth was measured every 3 days and calculated according to the following formula: volume = (length × width^2^)/2. After 28 days, the mice were anaesthetized with an overdose of barbiturate followed by cervical dislocation, and the tumor were removed from the mice and kept for weight measurement, hematoxylin–eosin staining (H&E) and immunofluorescence staining. The tumor sections were cut at a thickness of 10 μm with a microtome. Slides were stained with anti-active-caspase3 (1:200; 9664, CST) and anti-Ki67 (1:200; ab15580, Abcam) antibodies. Animal procedures were performed according to the Guide for the Care and Use of Laboratory Animals and were approved by the animal care and use committee of Shandong University.

### Statistical analysis

Statistical analysis was performed using SPSS (version 19.0). The values are presented as the mean ± SEM, and significance was set at *p* < 0.05. The statistical significance of the data was calculated using Student’s *t* test or one-way ANOVA, followed by the LSD post hoc test.

## Results

### Graphene oxide inhibits glioblastoma stem cell sphere formation and proliferation

**Fig. 1:**
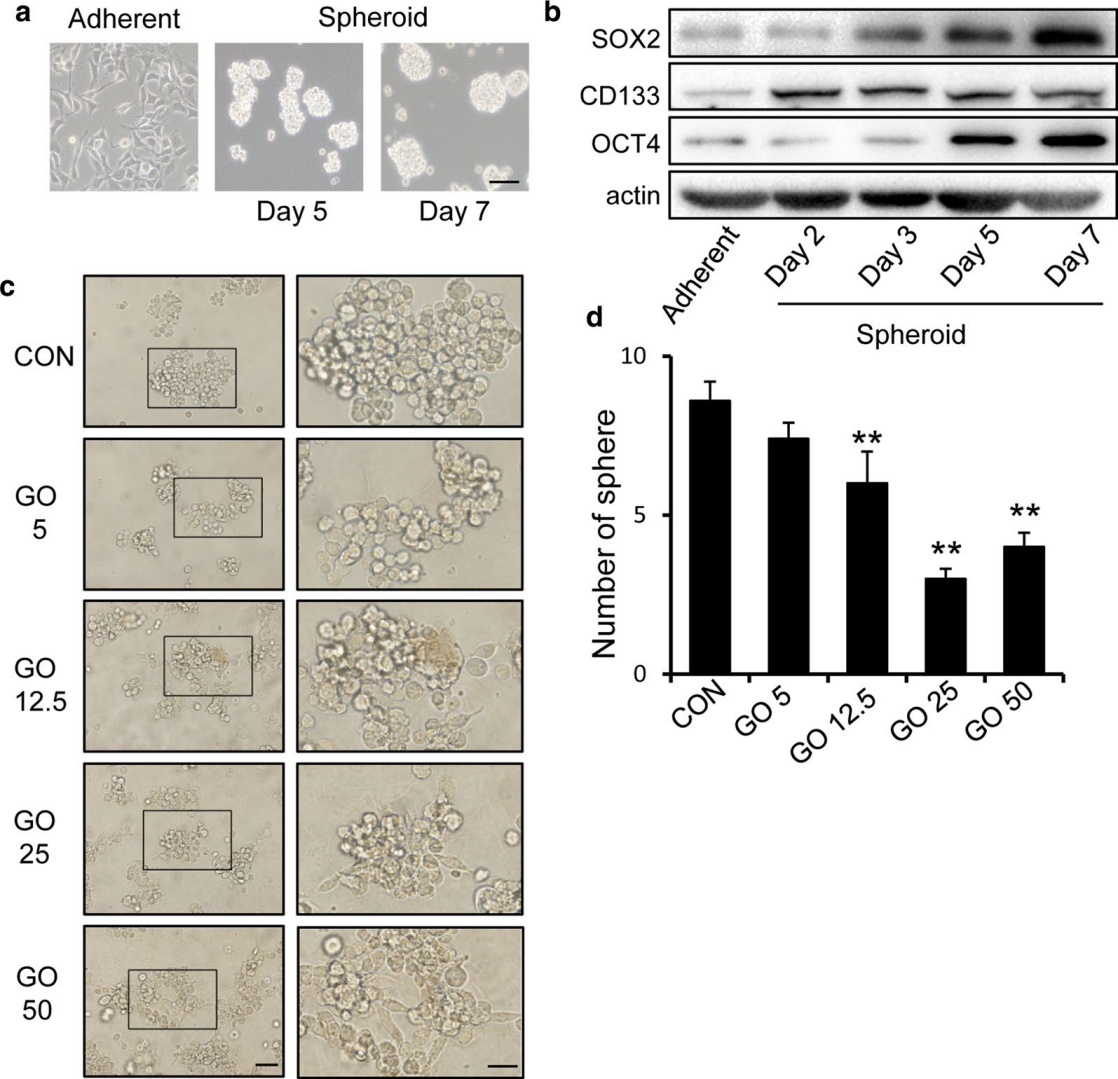
Graphene oxide influences the phenotypic properties and morphology of U87 GSCs. **a** U87 cells were cultured in a serum-free environment for 2–7 days. Sphere morphology was photographed using light microscopy. Scale bar = 100 μm. **b** The expression of SOX2, CD133 and OCT4 in glioblastoma stem-like cells was increased during different periods. **c** Morphological appearance of GSCs with or without GO treatment after 2 days. The GSC spheres subject to GO treatment showed adherent growth and some transformed to fusiform cells. Left: scale bar = 50 μm; right: scale bar = 20 μm. **d** The number of large GSC spheres (diameters larger than 50 μm) declined as the concentration of GO increased. The panel shows the number of spheres that were larger than 50 μm in different groups. The concentrations of GO were 5, 12.5, 25, 50 μg/ml. GSCs were counted in 5 random fields and data are expressed as mean ± SEM. *p < 0.05, **p < 0.01. Data represent the mean ± SEM of at least three independent experiments

To explore the effect of GO on GSCs, we used sphere cultures to induce spheroid body formation in U87 GBM cells (Fig. 1a) to enrich for GSCs [18]. The stem cell-related properties of the spheroids were further examined via the stemness-related marker, SOX2, OCT4, CD133 (Fig. 1b). Then, the GSCs were treated with GO at different concentrations for 2 days. Treatment with GO triggered dose dependent inhibition of GSC sphere formation from 12.5 μg/ml (Fig. 1c). GO treatment altered the sphere morphology of the GSCs, and resulted in a change from suspension to adherence and the appearance of fusiform cells when administered at doses of 25 μg/ml or higher. In addition, the number of GSC spheres larger than 50 μm decreased during GO treatment, as shown in the bar graph in Fig. 1d. The results indicated that GO inhibited sphere-forming capability and suggested the presence of a potential limit on GSC growth.

**Fig. 2:**
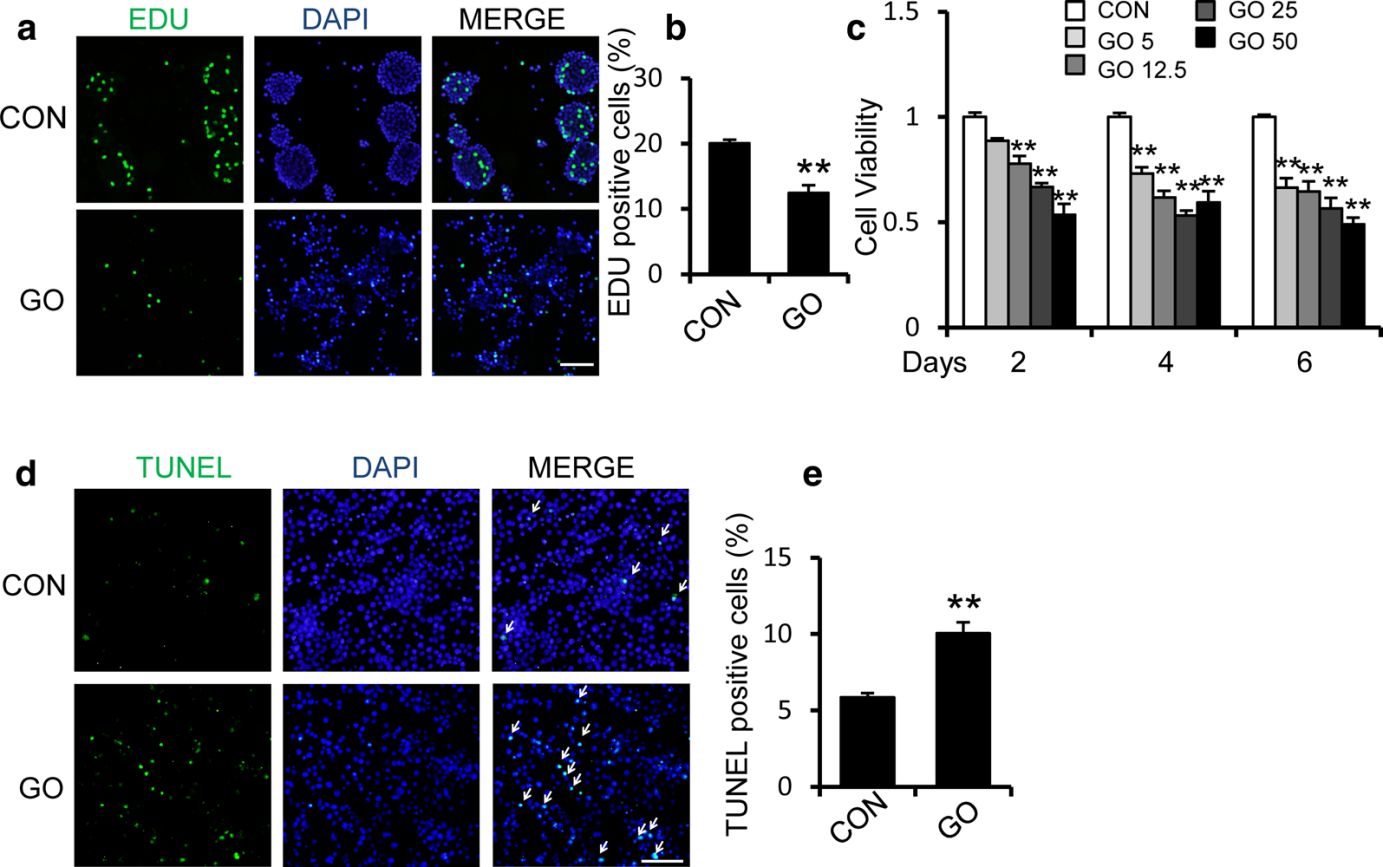
Graphene oxide inhibits the proliferation and survival of GSCs. **a**, **b** EdU staining indicated the cell proliferation capability of GSCs treated with 50 μg/ml GO for 2 days or that were untreated. The right panel shows the quantification of EdU-positive cells. Scale bar = 100 μm. **c** MTT assay indicated the cell viability of GSCs with or without treatment with different dosages of GO for 2, 4, and 6 days. **d**, **e** TUNEL staining of GSCs showed an increase in cell apoptosis after treatment with 50 μg/ml GO for 2 days. The right panel shows the quantification of the TUNEL-positive cells. Scale bar = 100 μm. *p < 0.05, **p < 0.01. Data represent the mean ± SEM of at least three independent experiments

We also assessed the effect of GO on GSC proliferation using an EdU incorporation assay, during which we observed that GSCs showed significant reductions in their proliferation rates, as indicated by an approximately 40% reduction in EdU-positive cells (Fig. 2a, b). The effect of GO on GSC viability was determined using an MTT assay that was conducted over 2 to 6 days. As shown in Fig. 2c, we also observed a dose-dependent inhibition of GSC viability in the presence of GO. Treatment with 50 μg/ml GO significantly increased GSC cell death, as observed via TUNEL staining (Fig. 2d–e).

Our preliminary results revealed that GO inhibited the growth of GSC spheres and altered sphere morphology in a concentration dependent manner.

### Graphene oxide inhibits the expression of stem cell markers and promotes the differentiation of GSCs

**Fig. 3:**
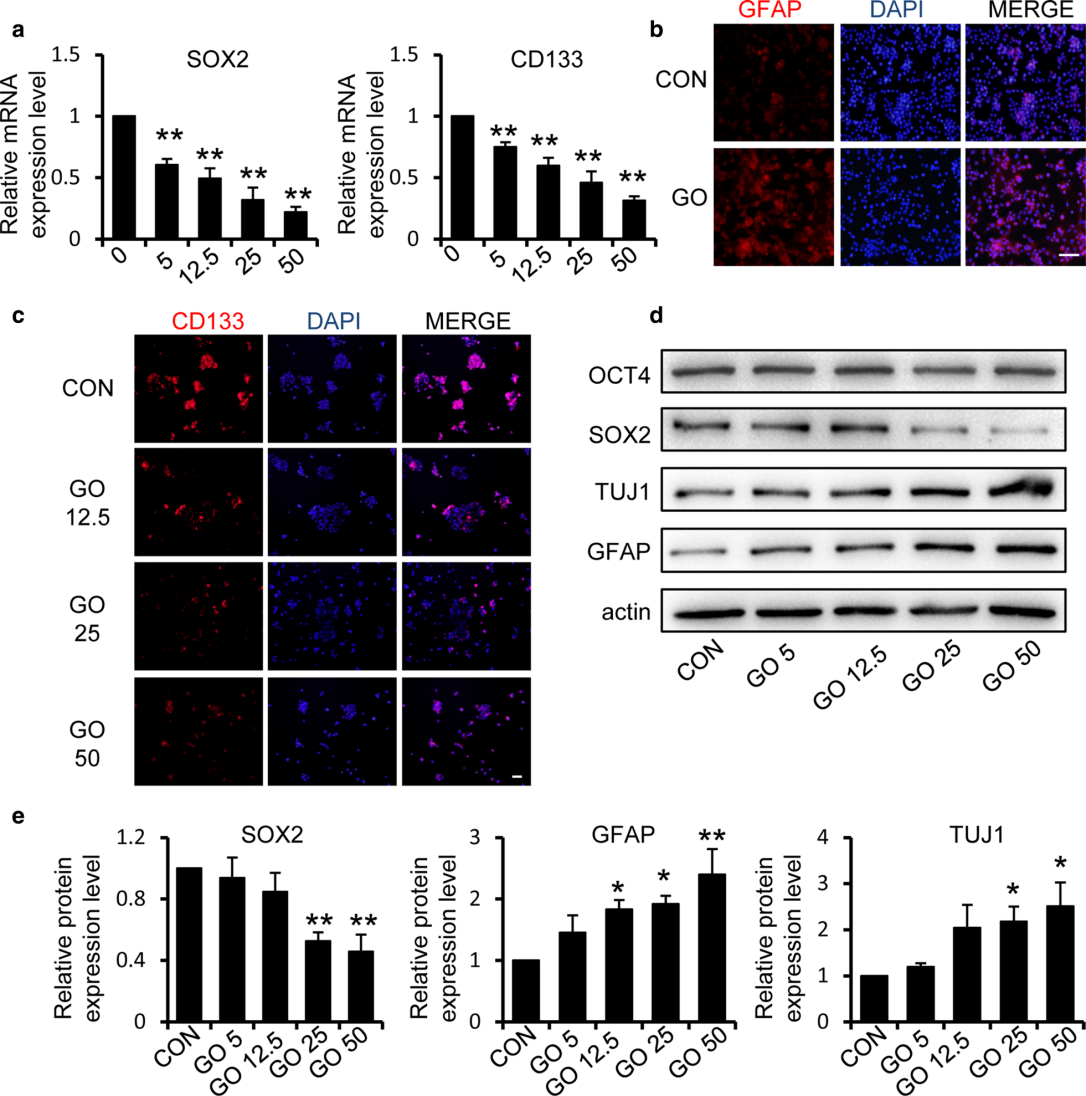
Graphene oxide reduces the expression of stem cell markers and promotes the differentiation of GSCs. **a** Quantification of the mRNA levels of stem cell markers SOX2 and CD133 in GSCs with or without treatment with GO. **b** The intracellular expression of the differentiation marker GFAP after treatment with 50 μg/ml GO was examined using immunofluorescence staining. Scale bar = 100 μm. **c** The expression level of the stem cell marker CD133 in cells treated with different concentrations of GO was detected by immunofluorescence staining. Scale bar = 50 μm. **d**, **e** Representative immunoblots and relative quantification of OCT4, SOX2, TUJ1 and GFAP in GSCs after treatment with 0, 5, 12.5, 25 and 50 μg/ml GO respectively. *p < 0.05, **p < 0.01. Data represent the mean ± SEM of at least three independent experiments

To further validate the observation that GO could reduce the stemness of GSCs, we examined several well-established stem cell markers (SOX2 and CD133) and differentiation markers (GFAP and β-III tubulin [TUJ1]). We first compared the variation in transcription factors in different groups treated with 5 μg/ml, 12.5 μg/ml, 25 μg/ml, and 50 μg/ml for 2 days. qPCR results showed that GSCs that were treated with GO expressed reduced mRNA levels of SOX2 and CD133 in a dose-dependent manner (Fig. 3a). Compared with the control group, the expression of GFAP was increased and that of CD133 was decreased in the GO group, as determined using immunofluorescent staining (Fig. 3b, c). In line with these results, western blotting indicated that GO induced a reduction in the expression of SOX2, while GO had no significant effect on the expression of OCT4 (Fig. 3d–e). We hypothesized that OCT4 may not be the key gene involved in the regulation of GSCs. The expression of differentiation markers GFAP and TUJ1 were significantly increased in a dose-dependent manner during treatment with GO (Fig. 3d, e).

**Fig. 4:**
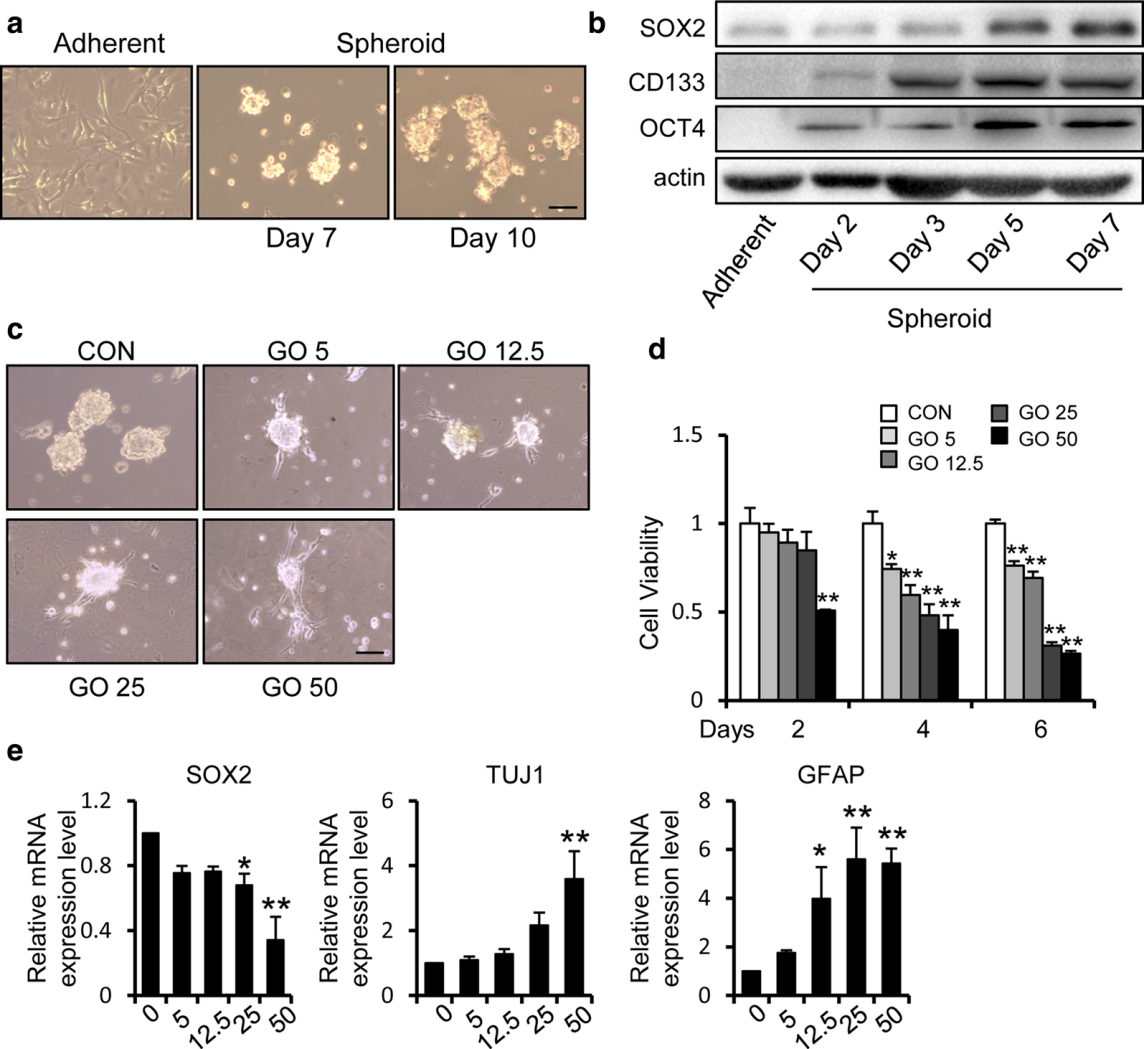
Graphene oxide inhibits the cell viability and promotes the differentiation of U251 GSCs. **a** U251 cells were cultured in a serum-free environment. Sphere morphology was photographed using light microscopy. Scale bar = 100 μm. **b** The expression of SOX2, CD133 and OCT4 in glioblastoma stem-like cells was increased during different periods. **c** Morphological appearance of U251 GSCs with or without treatment with GO for 2 days. The GSC spheres treated with GO showed adherent growth. Scale bar = 100 μm. **d** An MTT assay showed the cell viability of U251 GSCs with or without treatment with different dosages of GO for 2, 4, and 6 days. **e** Quantification of the mRNA levels of the stem cell markers SOX2 and differentiation markers (GFAP and TUJ1) in U251 GSCs with or without treatment with 0, 5, 12.5, 25 and 50 μg/ml GO respectively. *p < 0.05, **p < 0.01. Data represent the mean ± SEM of at least three independent experiments

To make our result more convincing, we also examined the effect of GO on U251 GSCs. We cultured another glioblastoma cell line, U251, and prepared GSCs using the previously described method (Fig. 4a). The stem cell-related properties of the spheroids were further examined via the stemness-related marker, SOX2, OCT4, CD133 (Fig. 4b). With the treatment of GO, the sphere morphology of U251 GSCs also reflected a state of adhesion and was characterized by the appearance of fusiform cells (Fig. 4c). We determined the influence of GO on the cell viability of U251 GSCs, during which we observed dose- and time-dependent inhibition of U251 GSC viability in the presence of GO (Fig. 4d). At the same time, the expression of SOX2 was decreased, and the expression of GFAP and TUJ1 was increased, during GO treatment (Fig. 4e). We also detected the effect of GO on primary GSC BG5 cells. The sphere morphology and MTT assay showed that GO inhibited the sphere formation and cell viability (Additional file 1: Figure S1A, B). Western blot showed that GO inhibited the expression of stem cell markers and promoted the differentiation of BG5 cells (Additional file 1: Figure S1C–G). We further examined whether the inhibitory effect of GO is specifically directed to stem cell-like properties or can target differentiated tumor cells. The EdU incorporation assay and MTT assay showed that GO also had inhibitory effect on the cell viability and proliferation of U87 and U251 tumor cells, but the inhibitory effect was weaker than that on GSCs (Additional file 2: Figure S2A–D).

Considering the effects of GO on two kinds of GSC cell lines and primary BG5 cells, it was suggested that GO decreased the expression of some stem cell markers and increased the expression of differentiation markers, which inhibited the tumor-propagating capacity of GSCs.

### Graphene oxide disturbs the cell cycle of GSCs and promotes the differentiation of GSCs via epigenetic mechanisms

**Fig. 5:**
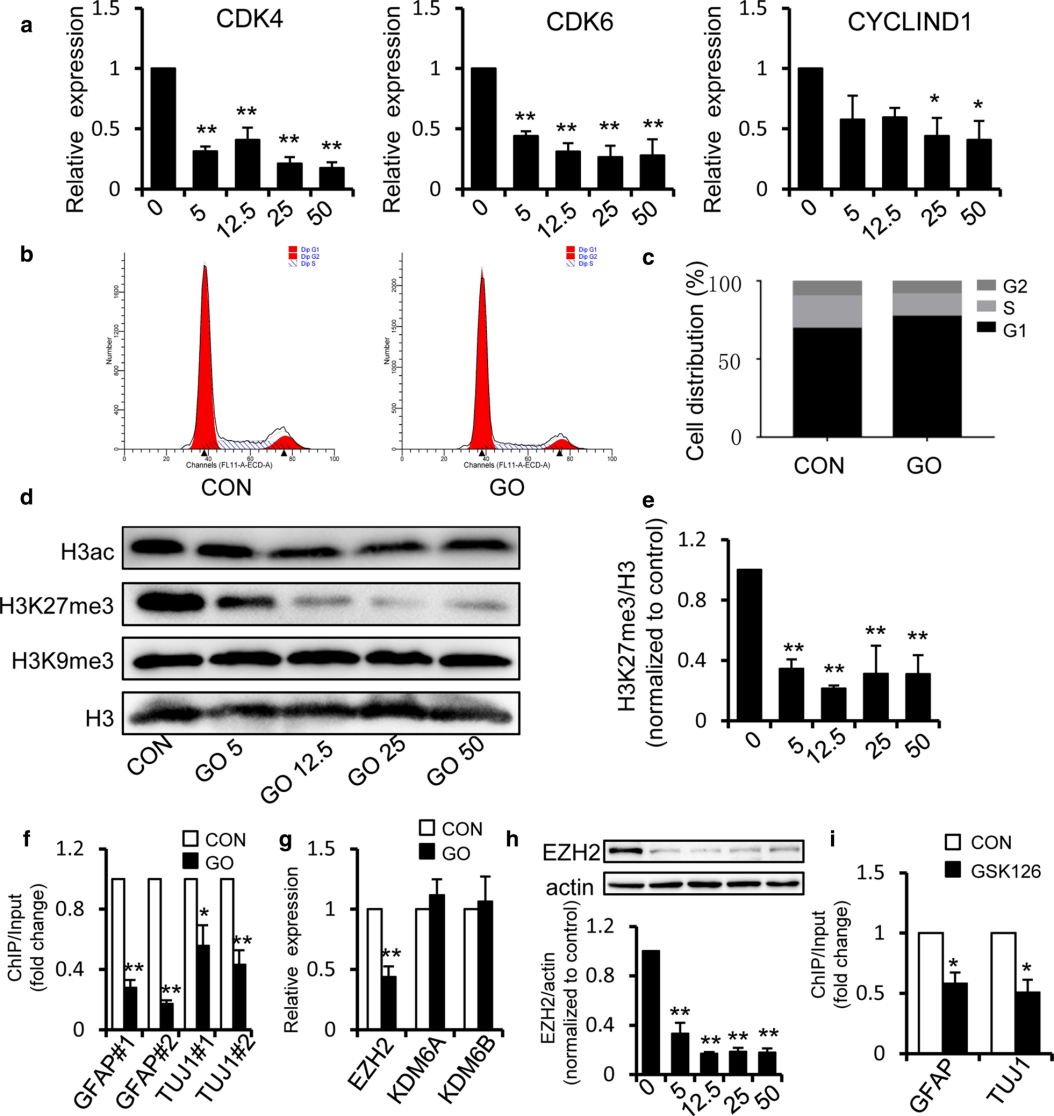
Graphene oxide regulates the cell cycle and promotes differentiation of GSCs via epigenetic mechanisms. **a** qRT-PCR showed the mRNA levels of the cell cycle regulators CDK4, CDK6 and CyclinD1 in GSCs after GO treatment. **b**, **c** GSCs treated with 50 μg/ml GO or control group were stained with PI and the cell cycle distribution was analyzed using flow cytometry. **d**, **e** Western blot analysis showed that the level of H3K27me3 was reduced after treatment with GO. **f** Chromatin immunoprecipitation showed that 50 μg/ml GO decreased the accumulation of H3K27me3 on the promoters of GFAP and TUJ1. Rabbit IgG was used as a negative control. The DNA in each ChIP sample was normalized based on the corresponding input sample. **g** The mRNA levels of the H3K27me3 methylase EZH2, and demethylases KDM6A and KDM6B in U87 GSCs after treatment with 50 μg/ml GO. **h** The protein expression level of the H3K27me3 regulator EZH2 was measured by western blot after treatment with 0, 5, 12.5, 25 and 50 μg/ml GO respectively. **i** Chromatin immunoprecipitation showed EZH2 inhibitor GSK126 decreased the accumulation of H3K27me3 on the promoters of GFAP and TUJ1 compared with control group. Rabbit IgG was used as a negative control. The DNA in each ChIP sample was normalized based on the corresponding input sample. *p < 0.05, **p < 0.01. Data represent the mean ± SEM of at least three independent experiments

Cell fate determination was coordinated with cell cycle regulation. The progression of the cell cycle is regulated by a series of events, such as those mediated by cyclin/cyclin-dependent kinase (CDK). The activated complex of CyclinD and CDK4/6 could phosphorylate the retinoblastoma protein and thus cause the arrest of the cell cycle in G_1_ [19]. To investigate whether GO promoted the differentiation of GSCs by regulating the cell cycle, we selected several significant regulators of the cell cycle and measured the mRNA expression levels by qPCR. Our results showed that the mRNA expression levels of CDK4, CDK6 and CyclinD1 declined during treatment with GO (Fig. 5a). To investigate whether GO induces cell cycle distribution in GSCs, flow cytometry analysis was conducted subsequent to GO treatment. As shown in Fig. 5b, c, GO treatment increased the percentage of G1 phase cells and decreased the percentage of S phase cells, indicating that GO did induce cell cycle arrest of GSCs. However, GO had no significant influence on the cell cycle of U87 and U251 tumor cells (Additional file 2: Fig. S2E), which is consistent with the previous research that GO and its derivatives didn’t disturb the normal cell cycle of U87 [20].

Given that epigenetic mechanisms contribute to clonogenic GSC growth, we determined the degree of acetylation and methylation of histone H3. The degree of total histone acetylation was analyzed using an acetyl-histone H3 antibody and found to be mostly unaffected in GO-treated GSCs (Fig. 5d). We measured histone methylation using H3K9me3 or H3K27me3 antibody and observed no discernable changes in the level of H3K9me3, while the level of H3K27me3 was decreased in GO-treated GSCs (Fig. 5d, e). Previous studies have indicated that H3K27me3 modification could influence the degree of chromosome enfoldment and even initiate tumorigenesis [21]. Our ChIP analysis, which used the H3K27me3 antibody, showed that GO treatment resulted in decreased levels of H3K27me3 in the promoters of differentiation-related genes, indicating that GO promoted the expression of differentiation-related genes via regulation of the H3K27me3 level (Fig. 5f). The levels of H3K27me3 are related to the roles of H3K27me3 methylase EZH2, and demethylases KDM6A and KDM6B [22]. We measured the levels of EZH2, KDM6A and KDM6B in GO-treated GSCs, and found that GO treatment did not alter the mRNA levels of KDM6A and KDM6B. However, the EZH2 level was significantly decreased (Fig. 5g). Then, the expression of EZH2 was measured by western blot, and we found that the expression of EZH2 was decreased in GO-treated GSCs, which is consistent with that observed for H3K27me3 (Fig. 5h). When EZH2 was inhibited using the EZH2 inhibitor GSK126, the levels of H3K27me3 in the promoters of GFAP and TUJ1 were reduced (Fig. 5i), which further demonstrating that GO inhibits the expression of GFAP and TUJ1 via the suppression of EZH2. Meanwhile, we also found that GO could downregulate the expression levels of H3K27me3 and EZH2 in U87 tumor cell, with a weaker degree (Additional file 2: Fig. S2F). Taken together, the inhibitory effects and mechanisms mediated by GO were not specifically directed to GSCs, but its effect is much stronger than that of tumor cells.

### Graphene oxide inhibits tumorigenesis in GSCs in vivo

**Fig. 6:**
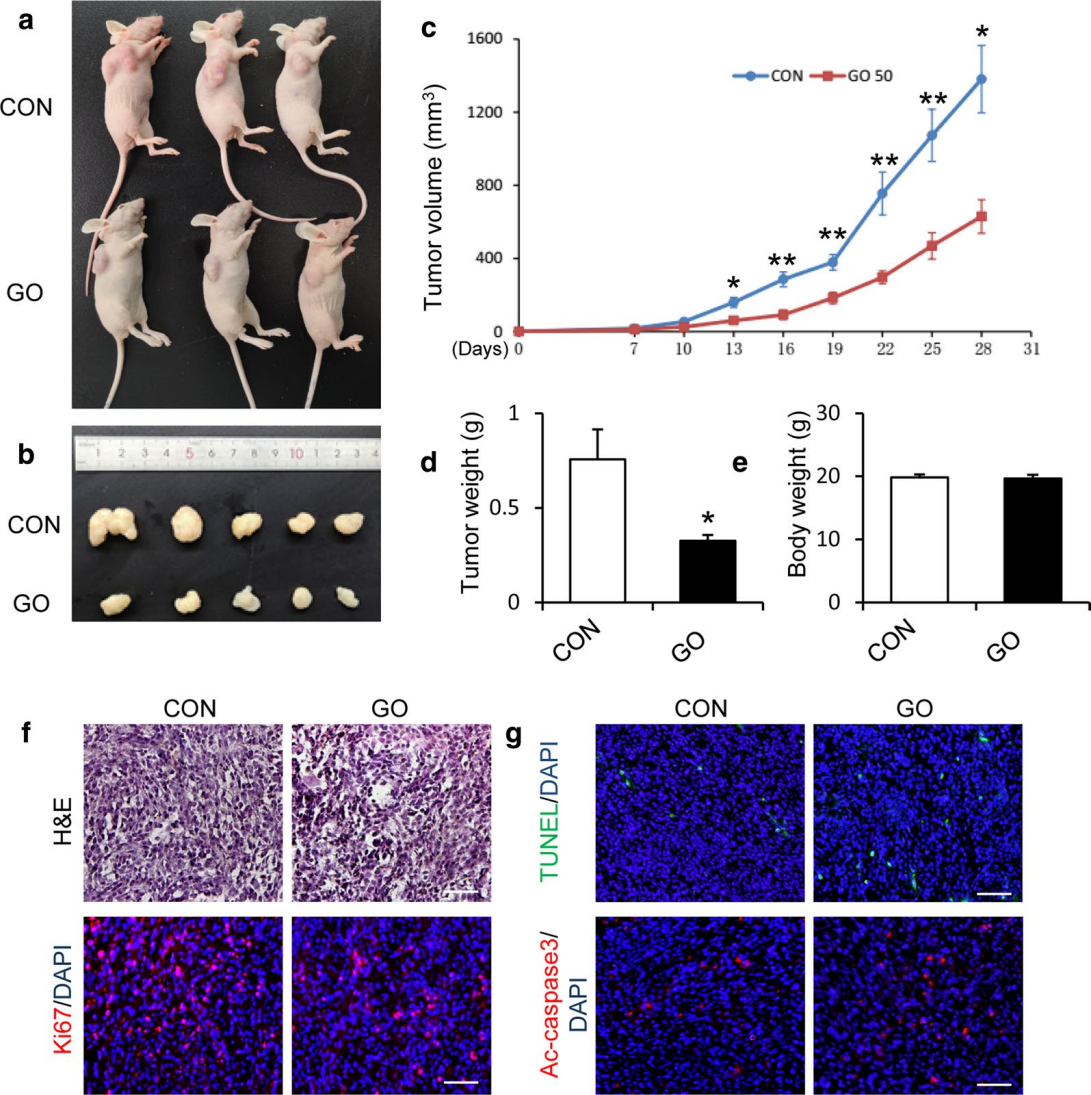
Graphene oxide inhibits the tumorigenesis of GSCs in a nude mouse model. **a**, **b** GSCs treated with 50 μm/ml GO or control GSCs were injected into nude mice (n = 5). The tumors formed in the GO-treated group were dramatically smaller than those formed in the control group. **c** The tumor volumes were quantified every 3 days after injection (n = 5). Data represent the mean ± SEM. **d**, **e** Tumor weights (**d**) and body weights (**e**) are presented as the mean ± SEM. **f** The tumor sections were performed with H&E staining and immunofluorescence staining using antibodies against ki-67. **g** Immunofluorescence staining of TUNEL (green) and active-caspase3 (red) were performed to evaluate the apoptosis of tumor cells. DAPI stains nuclei (blue). Scale bar = 50 μm. *p < 0.05, **p < 0.01. Data represent the mean ± SEM of at least three independent experiments

Based on the in vitro results, we next investigated the impact of GO in vivo. We inoculated control GSCs and GO-treated GSCs subcutaneously into nude mice. Compared with the tumors from the control group, the tumors derived from GO-treated GSCs were clearly smaller (Fig. 6a–c). The tumor weights of the GO-treated group were significantly reduced compared to those of the control group (Fig. 6d). However, the body weights of the mice were not significantly altered (Fig. 6e). Then the tumor sections were subjected to H&E staining and tumor tissue in the GO-treated group showed a decrease in the number of tumor cells (Fig. 6f). The immunofluorescence results indicated that the tumor tissue derived from the GO-treated GSCs displayed lower ki-67-positive cells than those formed from control group, demonstrating that GO inhibits the tumor cell growth in vivo (Fig. 6f). TUNEL and activated caspase3 (ac-caspase3) staining of comparable tumor sections in control and GO-treated group showed an increase in TUNEL^+^ and ac-caspase3^+^ cells (Fig. 6g). These findings indicate that GO increases the apoptosis of tumor cells in vivo. Collectively, these results indicated that GO might be an efficient agent that could be used to suppress tumor growth and malignancy in vivo.

**Fig. 7:**
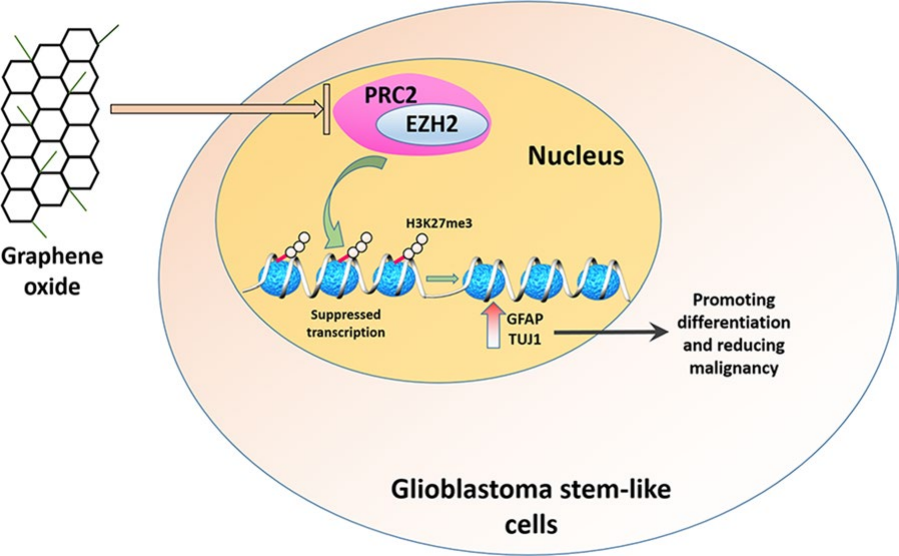
The molecular mechanism underlying GO-inhibited transcription in GSCs. GO downregulates the epigenetic methyl transferase EZH2 and enhances the expression of the differentiation related genes TUJ1 and GFAP. Data represent the mean ± SEM of at least three independent experiments

In summary, our results revealed that GO induced the cell cycle arrest, inhibited the expression of EZH2 and resulted in a decrease in H3K27me3 in the promoters of GFAP and TUJ1, which resulted in the differentiation of GSCs and reduced malignancy (Fig. 7).

## Discussion

The physicochemical properties provide graphene materials with outstanding potential in diverse biomedical fields, such as drug delivery and biomolecular detection [23, 24]. As a potential cancer therapeutic agent, GO could effectively inhibit the migration and invasion of human breast and prostate cancer cells and mouse melanoma cells [25]. The cancer stem cells were responsible for tumor relapse and treatment resistance, and became the focus of tumor therapy [26, 27]. In this study, we demonstrated that GO could inhibit the growth and formation of GSC spheres and reduce their malignant properties by inducing the differentiation of GSCs. Moreover, we found that GO treatment could regulate the expression levels of cell cycle regulators and induce the cell cycle arrest. GO appeared to reduce the levels of H3K27me3 in the promoters of differentiation-related genes via its regulation of EZH2. Finally, in vivo tumor model in mice showed the GO-treated GSCs exhibited decreased tumor growth.

In our study, GO’s inhibition of the growth and promotion of the differentiation of GSCs was thoroughly demonstrated. CD133 and SOX2 are largely utilized as cancer stem cell markers in GSCs [28]. In this study, for the first time, we showed that the expression levels of SOX2 and CD133 declined during treatment with different concentrations of GO, which also resulted in decreased cell proliferation and increased induction of apoptotic cell death. These results supported those of previous studies that showed that GO triggered cell death in cancer stem cells derived from other types of tumors [17]. Several studies have shown that GO is nontoxic in normal stem cells and could promote their differentiation [29]. GSCs are believed to represent a small subpopulation of highly tumorigenic cells with stem cell properties, which play significant roles in invasive tumor growth and therapy resistance. GFAP and TUJ1 were used as markers of differentiation in GSCs. Once the GSCs differentiated, their malignant properties declined. The present study first showed that GO induced GSCs to differentiate into astrocytes (GFAP-positive) and neurons (TUJ1-positive) both in U87, U251 GSC cell lines and primary GSC BG5 cell. Previously used cancer therapies could kill the majority of glioma cells; however, GSCs can maintain their proliferate properties and thereby result in glioma recovery after therapy. The consequences of promoting the differentiation of GSCs are to reduce their overall “stemness” and inhibit their proliferation. As GSCs are difficult to thoroughly eliminate, promoting the differentiation of GSCs could reduce the malignancy of a tumor. Most importantly, the in vivo results showed that GO inhibited cell growth in nude mice. Our study provides a novel approach that could be used to treat GSCs and reduce malignancy.

Although GSCs are becoming a focus of treatment, the way in which GO controls self-renewal and tumorigenesis in GSCs is unclear. Disturbance of the normal regulation of cell cycle progression is a key event in the development of cancer. The progression of the cell cycle from the G_1_ to the S phase is controlled by CDKs and cyclin complexes [30]. Our data showed that the expression of CDK4, CDK6 and CyclinD1 were reduced by GO. The downregulation of CDK4, CDK6 and CyclinD1 reflected the suppression of cell cycle progression. Moreover, flow cytometry analysis showed that GO induced cell cycle arrest. A previous study reported that graphene derivatives could promote apoptosis in osteosarcoma [31]. Our data showed that GO induced apoptosis in GSCs, as indicated by the TUNEL experiment. These results suggested that GO represses glioma growth by inducing apoptosis and cell cycle arrest in GSCs.

Recently, an accumulation of evidence has emerged to support the fact that epigenetic regulation, including histone modification, plays a crucial role in tumor progression. Histone modification, including histone methylation, is a major epigenetic mechanism that participates in tumor initiation and propagation. A previous study demonstrated that GSCs could be induced to differentiate as a result of epigenetic changes [32]. In our study, we found that H3K27me3 was a key modification that resulted from treatment with GO, while other modifications had little influence. H3K27me3 is modified by H3K27me3 methylase EZH2, and demethylases KDM6A and KDM6B. The expression of the H3K27me3-demethylase KDM6A is reduced in stem-like subpopulations of mammary cell lines and stem cell-enriched triple-negative breast cancers, indicting the significance of H3K27me3 in cancer stem cell [33]. Our data showed that both the expression of EZH2 and H3K27me3 modification were reduced during GO treatment in a concentration-dependent manner, while the expression of KDM6A and KDM6B were not regulated by GO treatment. Through the ChIP experiment that was performed for H3K27me3, we revealed that the differentiation markers GFAP and TUJ1 were regulated by EZH2/H3K27me3 and, ultimately, by GO. It is likely that GO regulated the expression of EZH2 and then influenced the modification of H3K27me3. However, we have only verified the epigenetic mechanism of GO in U87 GSC, and whether it works in other GSCs remains to be further explored. In addition, we also found that GO could inhibit the cell viability and suppress the expression levels of H3K27me3 and EZH2 in U87 tumor cell, with a lower sensitivity than that of GSCs. This further proves that GO could affect the epigenetic modification of cells. However, GO was not well-absorbed by cells [34]. The exact mechanism that underlies the effect of GO on epigenetic regulation is still not clear. It has been demonstrated that surface contact could influence receptors on the cell surface, thereby altering cell activity. GO could regulate the NSC cytoskeleton to promote differentiation or impair extracellular adhesion, which would decrease migration in glioblastoma [35, 36]. In our opinion, this hypothesis requires further investigation.

## Conclusions

The present study demonstrated that GO could effectively inhibit the proliferation and induce the differentiation of GSCs by reducing the self-renewal, decreasing stemness-related gene expression and increasing differentiation-related gene expression. Its therapeutic effects may be related to the induction of apoptosis, cell cycle arrest and the EZH2-mediated epigenetic modification of H3K27me3 in GSCs. However, further studies are needed to confirm its therapeutic effect in clinic. These studies will aid in elucidating the mechanisms utilized by GO and thereby lead to the development of an effective method for targeting GSCs.

## Supplementary information

**Additional file 1: Fig. S1.** (A) Morphological appearance of primary BG5 GSCs with or without the treatment of GO for 2 days. The spheres of GSCs with GO treatment were smaller. Scale bar = 100 μm. (B) MTT assay showed the cell viability of BG5 GSCs with or without treatment of different dosage GO for 2, 4, 6 days. (C–G) Representative immunoblots and relative quantification of SOX2, CD133, TUJ1 and GFAP in BG5 GSCs after treatment with 0, 5, 12.5, 25 and 50 μg/ml GO respectively. *p < 0.05, **p < 0.01. Data represent the mean ± SEM of at least three independent experiments.

**Additional file 2: Fig. S2.** (A) EdU staining indicated the cell proliferation capability of U87 tumor cell treated with 50 μg/ml GO for 2 days or that were untreated. The right panel shows the quantification of EdU-positive cells. Scale bar = 100 μm. (B) MTT assay showed the cell viability of U87 tumor cell with or without treatment of different dosage GO for 2, 4, 6 days. (C) EdU staining indicated the cell proliferation capability of U251 tumor cell treated with 50 μg/ml GO for 2 days or that were untreated. The right panel shows the quantification of EdU-positive cells. Scale bar = 100 μm. (D) MTT assay showed the cell viability of U251 tumor cell with or without treatment of different dosage GO for 2, 4, 6 days. (E) U87 or U251 tumor cells treated with 50 μg/ml GO or control group were stained with PI and the cell cycle distribution was analyzed using flow cytometry. (F) Western blot analysis showed that the levels of H3K27me3 and EZH2 was reduced in U87 tumor cell after treatment with GO. *p < 0.05, **p < 0.01. Data represent the mean ± SEM of at least three independent experiments.

## Data Availability

The datasets used and/or analyzed during the current study are available from the corresponding author on reasonable request.

## Abbreviations

GSCs: Glioblastoma stem-like cells
GBM: Glioblastoma multiforme
GO: Graphene oxide
PRC2: Polycomb repressive complex 2
H3K27me3: Trimethylation of lysine 27 on histone H3
EZH2: The enhancer of zeste 2
MTT: Dimethyl thiazolyldiphenyl tetrazolium
TUNEL: TdT-mediated dUTP nick end labeling
PI: Propidium iodide
H&E: Hematoxylin–eosin staining
CDK: Cyclin/cyclin-dependent kinase
