# Supplementary figures and images for: Graphene oxide suppresses the growth and malignancy of glioblastoma stem cell-like spheroids via epigenetic mechanisms
Source: J Transl Med. 2020 May 14;18:200. doi: 10.1186/s12967-020-02359-z (PMC7227195; doi:10.1186/s12967-020-02359-z)

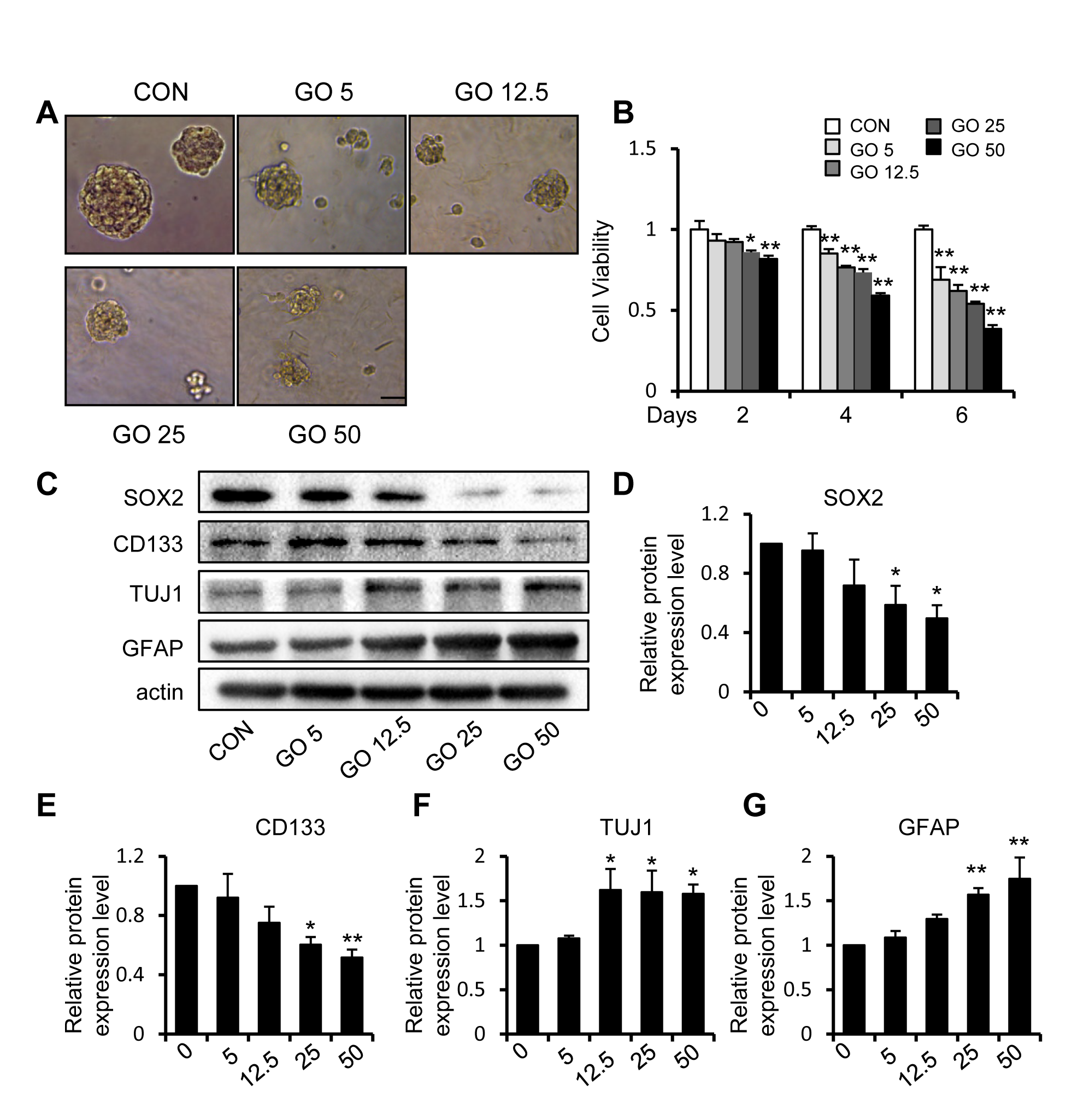

Supplement: Supplementary file 1 — Additional file 1: Fig. S1. (A) Morphological appearance of primary BG5 GSCs with or without the treatment of GO for 2 days. The spheres of GSCs with GO treatment were smaller. Scale bar = 100 μm. (B) MTT assay showed the cell viability of BG5 GSCs with or without treatment of different dosage GO for 2, 4, 6 days. (C–G) Representative immunoblots and relative quantification of SOX2, CD133, TUJ1 and GFAP in BG5 GSCs after treatment with 0, 5, 12.5, 25 and 50 μg/ml GO respectively. *p < 0.05, **p < 0.01. Data represent the mean ± SEM of at least three independent experiments. [file 12967_2020_2359_MOESM1_ESM.tif]

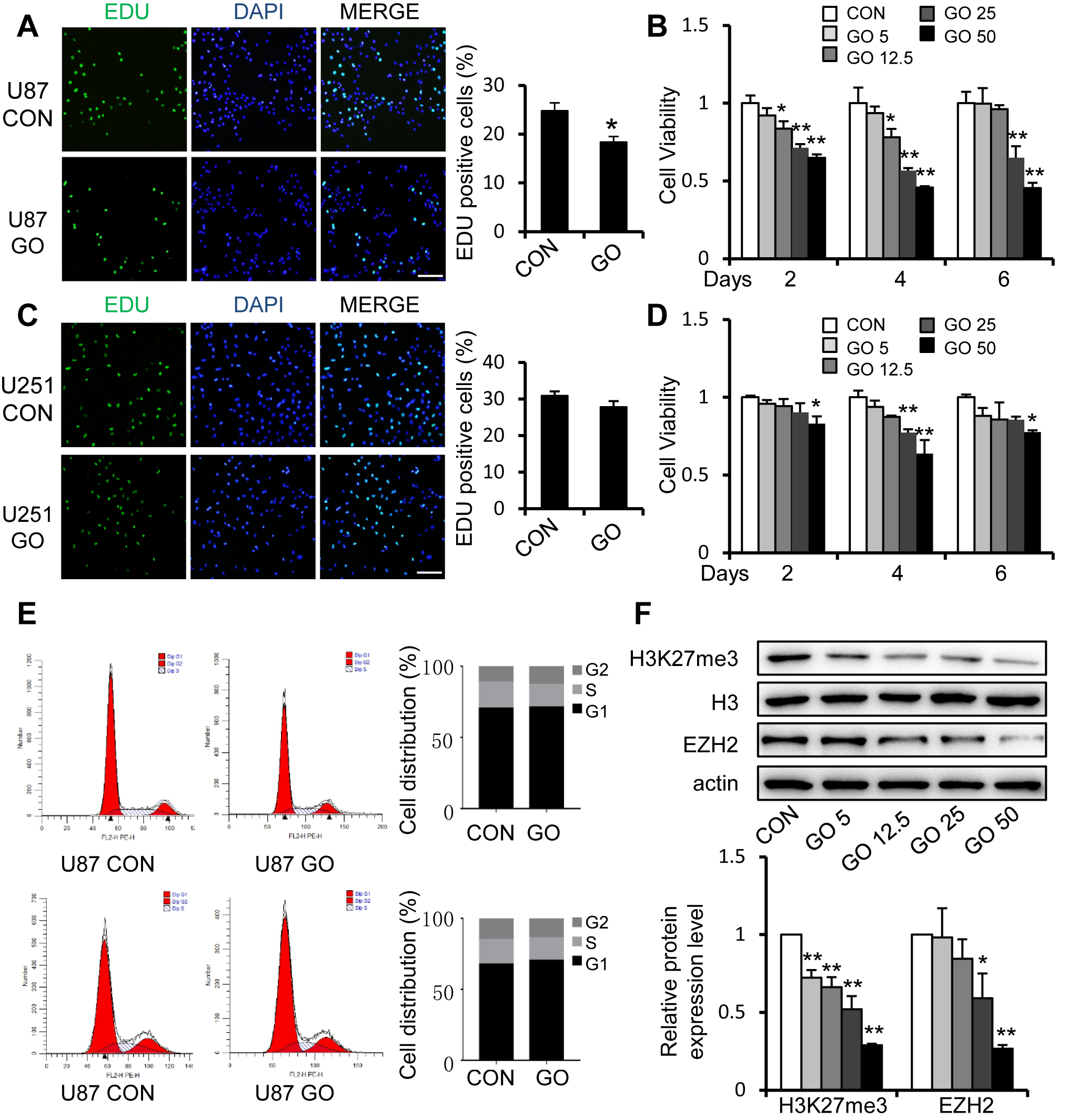

Supplement: Supplementary file 2 — Additional file 2: Fig. S2. (A) EdU staining indicated the cell proliferation capability of U87 tumor cell treated with 50 μg/ml GO for 2 days or that were untreated. The right panel shows the quantification of EdU-positive cells. Scale bar = 100 μm. (B) MTT assay showed the cell viability of U87 tumor cell with or without treatment of different dosage GO for 2, 4, 6 days. (C) EdU staining indicated the cell proliferation capability of U251 tumor cell treated with 50 μg/ml GO for 2 days or that were untreated. The right panel shows the quantification of EdU-positive cells. Scale bar = 100 μm. (D) MTT assay showed the cell viability of U251 tumor cell with or without treatment of different dosage GO for 2, 4, 6 days. (E) U87 or U251 tumor cells treated with 50 μg/ml GO or control group were stained with PI and the cell cycle distribution was analyzed using flow cytometry. (F) Western blot analysis showed that the levels of H3K27me3 and EZH2 was reduced in U87 tumor cell after treatment with GO. *p < 0.05, **p < 0.01. Data represent the mean ± SEM of at least three independent experiments. [file 12967_2020_2359_MOESM2_ESM.tif]
